# Supplementary material for: Coevolutionary dynamics of viruses and their defective interfering particles
Source: PLoS Comput Biol. 2026 May 20;22(5):e1014300. doi: 10.1371/journal.pcbi.1014300 (PMC13232958; doi:10.1371/journal.pcbi.1014300)
Supplement: S1 Table — Confusion matrix comparing outcome classifications at T = 200 versus T = 400 for all 10,000 LHS simulations. Overall concordance: 9,286/10,000 (92.9%). (PDF) [file pcbi.1014300.s003.pdf]

S1 Table. Convergence validation.

Classification at $T = 200$	Classification at $T = 400$				Total
	Chase	Coexistence	DIP Extinction	Coextinction	
Chase	1,782	531	0	0	2,313
Coexistence	168	7,212	4	0	7,384
DIP Extinction	0	11	12	0	23
Coextinction	0	0	0	280	280
**Total**	1,950	7,754	16	280	10,000
