# Supplementary figures and images for: Coevolutionary dynamics of viruses and their defective interfering particles
Source: PLoS Comput Biol. 2026 May 20;22(5):e1014300. doi: 10.1371/journal.pcbi.1014300 (PMC13232958; doi:10.1371/journal.pcbi.1014300)

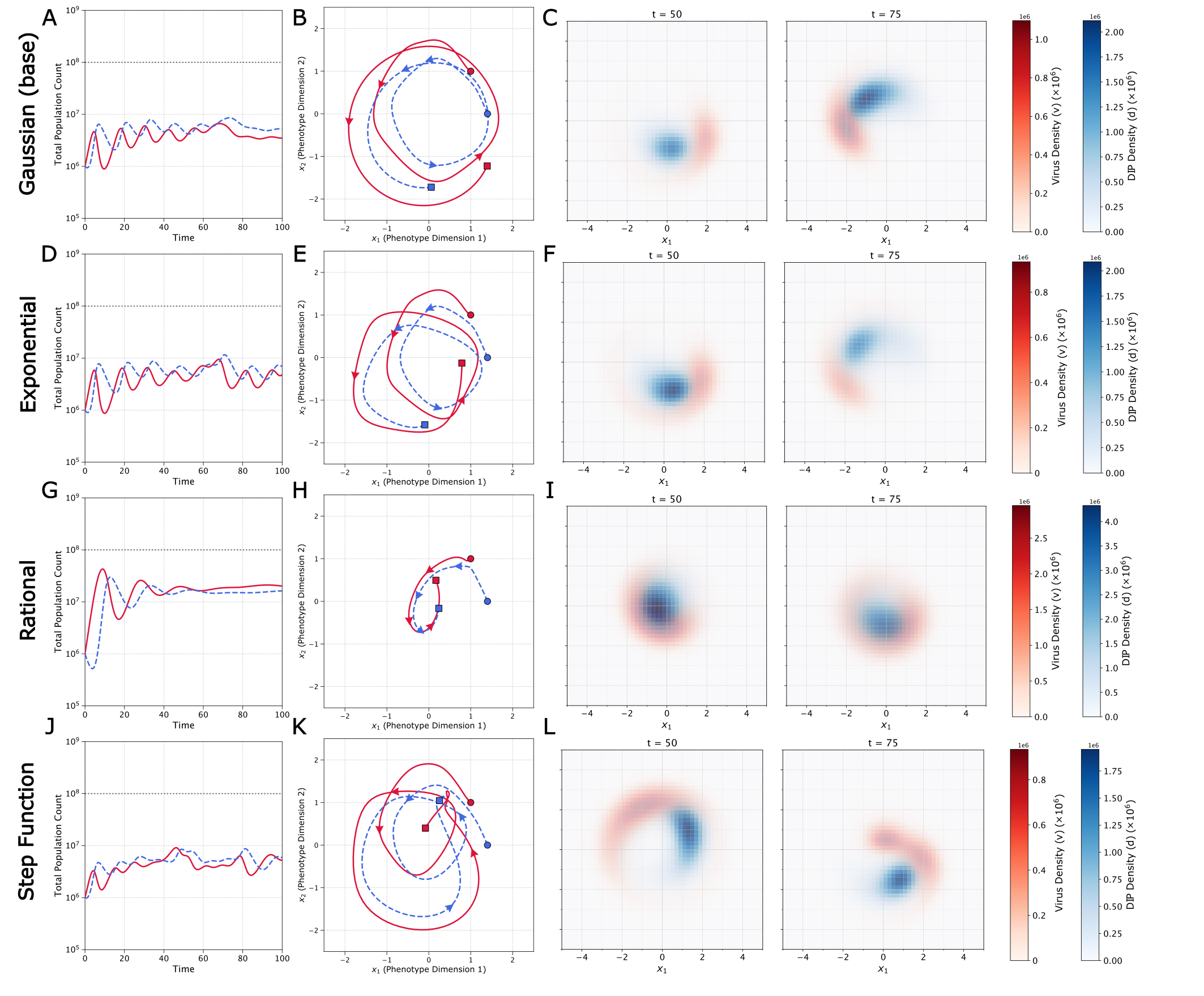

Supplement: S2 Fig — The main text model uses a Gaussian interference kernel (e−‖𝐱‖22σ2), to capture the intuition that more similar DIPs will better be able to interfere with the virus. However, we relax this assumption and test several other kernels. These include exponential (e−‖x‖σ, slower decay) (d-f), rational (11+‖x‖, much slower decay) (g-i), and step function (1 if ‖x‖<σ, else 0) (j-l). For the most part, these different kernels yield distinct but not disparate trajectories, suggesting that the model is robust to the choice of interference kernel. (TIFF) [file pcbi.1014300.s005.tiff]
