# Supplementary material for: Coevolutionary dynamics of viruses and their defective interfering particles
Source: PLoS Comput Biol. 2026 May 20;22(5):e1014300. doi: 10.1371/journal.pcbi.1014300 (PMC13232958; doi:10.1371/journal.pcbi.1014300)
Supplement: S1 Text — Defines the dimensionless variables and parameter groups used to rescale the dimensional model (Eqs. 1–2), provides the full dimensionless system (Eqs. S1–S2), and summarizes all dimensionless parameters in a reference table. All parameter values reported in the main text are these dimensionless groups. (PDF) [file pcbi.1014300.s007.pdf]

# S1 Text. Nondimensionalization of the model equations

To reduce the number of free parameters, we introduce dimensionless variables

$$\begin{aligned}\tilde{t} &= r_V\, t\\ \tilde{\mathbf{x}} &= \frac{\mathbf{x}}{\sigma}\\ \tilde{v}(\tilde{\mathbf{x}},\tilde{t}) &= \sigma^2\, v(\mathbf{x},\, t)\\ \tilde{d}(\tilde{\mathbf{x}},\tilde{t}) &= \sigma^2\, d(\mathbf{x},\, t)\end{aligned}$$

so that time is measured in units of the viral replication cycle and phenotypic distance in standard deviations of the Gaussian kernel. The carrying capacity $K$ retains its dimensional value in particles for biological interpretability. The rescaling absorbs $r_V$ and $\sigma$ into dimensionless parameter groups:

$$\tilde{\mu} = \frac{\mu}{r_V \sigma^2}, \qquad \tilde{\alpha} = \frac{\alpha\, \sigma^2}{r_V}, \qquad \tilde{\beta} = \frac{\beta\, \sigma^2}{r_V},$$
$$\tilde{\eta} = \frac{\eta}{r_V}, \qquad \tilde{\kappa} = \frac{\kappa}{r_V \sigma^2}, \qquad \tilde{\gamma} = \frac{\gamma}{r_V}.$$

Substituting into Eqs.1–2 and dividing through by $r_V$ yields the dimensionless system:

$$\begin{aligned}\partial_{\tilde{t}}\, \tilde{v}(\tilde{\mathbf{x}},\, \tilde{t}) &= \tilde{\mu}\, \tilde{\nabla}^2 \tilde{v}(\tilde{\mathbf{x}},\, \tilde{t}) - \tilde{\gamma}\, \tilde{v}(\tilde{\mathbf{x}},\, \tilde{t})\\ &\quad + \Big(1 - \tilde{\alpha}\, |\tilde{\mathbf{x}}|^2 - \tilde{\beta}\, \big|\tilde{\mathbf{x}} - \bar{\tilde{\mathbf{x}}}_V(\tilde{t})\big|^2 - \tilde{\eta} - \tilde{I}_V[\tilde{d}](\tilde{\mathbf{x}},\, \tilde{t})\Big)\, L(\tilde{t})\, \tilde{v}(\tilde{\mathbf{x}},\, \tilde{t})\end{aligned} \tag{S1}$$

$$\begin{aligned}\partial_{\tilde{t}}\, \tilde{d}(\tilde{\mathbf{x}},\, \tilde{t}) &= \tilde{\mu}\, \tilde{\nabla}^2 \tilde{d}(\tilde{\mathbf{x}},\, \tilde{t}) - \tilde{\gamma}\, \tilde{d}(\tilde{\mathbf{x}},\, \tilde{t})\\ &\quad + \Big(\tilde{I}_D[\tilde{v}](\tilde{\mathbf{x}},\, \tilde{t})\, \tilde{d}(\tilde{\mathbf{x}},\, \tilde{t}) + \tilde{\eta}\, \tilde{v}(\tilde{\mathbf{x}},\, \tilde{t})\Big)\, L(\tilde{t})\end{aligned} \tag{S2}$$

where the interference terms are

$$\tilde{I}_V[\tilde{d}](\tilde{\mathbf{x}},\, \tilde{t}) = \tilde{\kappa} \int_{\mathbb{R}^2} \tilde{G}(\tilde{\mathbf{x}} - \tilde{\mathbf{x}}')\, \tilde{d}(\tilde{\mathbf{x}}',\, \tilde{t})\, d^2\tilde{\mathbf{x}}' \tag{S3}$$

$$\tilde{I}_D[\tilde{v}](\tilde{\mathbf{x}},\, \tilde{t}) = \tilde{\kappa} \int_{\mathbb{R}^2} \tilde{G}(\tilde{\mathbf{x}} - \tilde{\mathbf{x}}')\, \tilde{v}(\tilde{\mathbf{x}}',\, \tilde{t})\, d^2\tilde{\mathbf{x}}' \tag{S4}$$

and the logistic factor is

$$L(\tilde{t}) = 1 - \frac{V(\tilde{t}) + D(\tilde{t})}{K},$$

In the main text, tildes are dropped for readability; all reported parameter values are the dimensionless groups defined above. The table below summarizes the full set of dimensionless and retained parameters.

**Nondimensionalized model parameters and functions.**

| Symbol | Definition | Description |
|---|---|---|
| **Dimensionless parameters** | | |
| $\tilde{\mu}$ | $\dfrac{\mu}{r_V \sigma^2}$ | Mutation rate (phenotypic diffusion) |
| $\tilde{\alpha}$ | $\dfrac{\alpha\,\sigma^2}{r_V}$ | Strength of selection toward the origin |
| $\tilde{\beta}$ | $\dfrac{\beta\,\sigma^2}{r_V}$ | Strength of the aggregation penalty |
| $\tilde{\eta}$ | $\dfrac{\eta}{r_V}$ | Rate of *de novo* DIP generation |
| $\tilde{\gamma}$ | $\dfrac{\gamma}{r_V}$ | Background removal (decay + dilution) rate |
| **Reduced-dimension parameter** | | |
| $\tilde{\kappa}$ | $\dfrac{\kappa}{r_V \sigma^2}$ | Per-particle interference strength (particles$^{-1}$; retains this unit because populations are kept in particles) |
| **Dimensionless functions** | | |
| $\tilde{G}(\tilde{\mathbf{x}})$ | $\dfrac{1}{2\pi}\, e^{-\lVert\tilde{\mathbf{x}}\rVert^2/2}$ | Standard-normal Gaussian kernel |
| $\tilde{I}_V[\tilde{d}](\tilde{\mathbf{x}},\, \tilde{t})$ | $\tilde{\kappa} \displaystyle\int \tilde{G}(\tilde{\mathbf{x}} - \tilde{\mathbf{x}}')\, \tilde{d}(\tilde{\mathbf{x}}',\, \tilde{t})\, d^2\tilde{\mathbf{x}}'$ | Interference cost to virus |
| $\tilde{I}_D[\tilde{v}](\tilde{\mathbf{x}},\, \tilde{t})$ | $\tilde{\kappa} \displaystyle\int \tilde{G}(\tilde{\mathbf{x}} - \tilde{\mathbf{x}}')\, \tilde{v}(\tilde{\mathbf{x}}',\, \tilde{t})\, d^2\tilde{\mathbf{x}}'$ | Interference benefit to DIP |
| $L(\tilde{t})$ | $1 - \big(V(\tilde{t}) + D(\tilde{t})\big)/K$ | Logistic population cap |
| $\bar{\tilde{\mathbf{x}}}_V(\tilde{t})$ | $\dfrac{1}{V(\tilde{t})} \displaystyle\int \tilde{\mathbf{x}}\, \tilde{v}(\tilde{\mathbf{x}},\, \tilde{t})\, d^2\tilde{\mathbf{x}}$ | Mean virus phenotype |
| $\bar{\tilde{\mathbf{x}}}_D(\tilde{t})$ | $\dfrac{1}{D(\tilde{t})} \displaystyle\int \tilde{\mathbf{x}}\, \tilde{d}(\tilde{\mathbf{x}},\, \tilde{t})\, d^2\tilde{\mathbf{x}}$ | Mean DIP phenotype |
| **Retained dimensional quantities** | | |
| $K$ | — | Carrying capacity (particles) |
| $V(\tilde{t}),\ D(\tilde{t})$ | — | Total virus and DIP populations (particles) |
