# Supplementary material for: Coevolutionary dynamics of viruses and their defective interfering particles
Source: PLoS Comput Biol. 2026 May 20;22(5):e1014300. doi: 10.1371/journal.pcbi.1014300 (PMC13232958; doi:10.1371/journal.pcbi.1014300)
Supplement: Attachment — Submitted filename: PCOMPBIOL_Review_Report.pdf [file pcbi.1014300.s008.pdf]

Review report of manuscript PCOMPBIOL-D-25-02183

'Coevolutionary dynamics of viruses and their defective interfering particles'
submitted for publication in PLOS Computational Biology

Manuscript summary

The authors propose a mathematical model of eco-evolutionary dynamics of a virus and its defective interfering particles (DIPs), both structured by an abstract phenotypic trait determining their fitness. This choice is made since DIPs, which are generated from the virus, may only reproduce when co-infecting a cell with a virus of similar phenotype, thus interfering with virus replication, ultimately providing a promising therapeutic strategy. The authors take into account the ability of the virus and DIPs to undergo mutations, allowing virus to escape DIP interference, and accounting for fitness trade-offs. Via numerical simulations and a global sensitivity analysis, the authors use this theoretical framework to show how accounting for mutations, competition and trade-offs can effectively explain the dynamics of transient resistance of the virus to DIPs that is observed empirically, as well as the so-called Red Queen chase typically captured by models prescribing coevolutionary feedback, and explore how model parameters impact co-evolutionary outcomes (coevolutionary chase, coexistence, DIP extinction or coextinction). The paper concludes with a discussion on potential model modifications, as well as experimental and theoretical perspectives. All code is publicly available on github.

Recommendation

The paper is generally well-written, providing a clear explanation of the biological problem, motivation for the study, literary context and originality. It could however benefit from more detail in the presentation of the mathematical quantities studied and methods used, along with more references on phenotype-structured PDEs which are very popular in other co-evolutionary contexts. The interdisciplinary aspect and certain biological concepts could also be better highlighted.

While I was initially sceptical of the ability of the model to draw significant biological conclusions given the level of abstraction of the phenotypic trait in such specific context, I have to say that the authors still manage to draw strong theoretical conclusions which can provide food for thought in experimental settings, always making an effort to link results back to specific biological features.

For these reasons, I recommend the manuscript for publication after several (minor) revisions, and I detail my suggestions below. I am certain this will be a valuable addition to the literature.

More detailed comments

Major points

1. I found the model description tricky to follow since the authors introduce individual terms in the PDEs before introducing the PDEs. This was only made easier by the fact that I am very familiar with phenotype-structured PDE models, and I think a change in presentation may better guide readers interested in the application who may not be too familiar with this type of models.

2. In figure 1 the authors show an experimental setup: this is interesting and I think it should be better introduced in the manuscript, together with appropriate references. For instance I am not sure whether it is an illustration of the abstraction level of the model, or an actual setup used in wet lab experiments. If the latter is the case, than this is a point in favour for the paper which would better tie together the biological conclusions. In fact, I think the use of titled paragraphs in the discussion could be useful, with one dedicated to the interdisciplinary conclusions and perspectives.

3. A critical point is that the authors in the results section often discuss the numerical outcomes talking about "stable" dynamics reached by the system, e.g. line 261 "a stabe, cyclical pursuit". How is stability determined numerically? For instance while in fig 3E it looks like a steady dynamics has been reached, the associated population dynamics in fig 3D do not appear to have stabilised, suggesting this might be a transient behaviour. The authors should better indicate how certain conclusions are quantitatively determined.

4. On a similar note, the authors should better state how an "outcome" is determined. For instance in the sensitivity analysis, are they plotting the observed dynamics at a certain fixed time? If so, how is this time chosen? Indeed the convergence speed to a steady outcome may change as parameter values change. How is convergence to an outcome determined numerically? The same question holds for the results of fig 7: what is meant by "final" virus population? This is even more vague if the system stabilises to a cyclic solution of coevolutionary chase, in which case population numbers will fluctuate in time. What summary statistic is being plot exactly? What were the initial conditions for these simulations? This improved precision is required throughout the results section.

5. The (mathematical) definition of "phenotypic distance" should be explicitly stated, indicating what summary statistic is being plot in fig 4b. Also, here the authors use the notation $\bar{x}_D$ but never introduced it before – this could be done in (9).

6. In fig 6 and Cyclical escape: it is not clear how "resistance" is defined and computed. A (mathematical) definition of the quantity plot is necessary. I find the visualisation in this figure very confusing, especially since we are looking at a function of time and time.... If I have inferred correctly, this is again the distance between $\bar{x}_v(t)$ and $\bar{x}_d(t)$, seen as a function of $\bar{x}_v(t)$ and $\bar{x}_d(t)$ which vary on the x- and y-axis respectively. I understand that the authors are choosing to compare "resistance" (i.e. phenotypic distance?) of the virus to DIPs at the same time or in the past or future to reproduce this concept of transient resistance found in the experimental paper cited. What is not clear to me is why this is relevant: since the dynamics of virus and DIPs are coupled, we will never actually have to deal with the current phenotype of the virus interacting with the past or future phenotype of DIPs. I am certain DePolo et al. gave a relevant motivation for this, which should be indicated, along with how the quantities plot in fig 6C were defined, obtained and measured experimentally.

7. The results of simulations plot in fig 7 are often referring to how close the trait of the virus is to the origin, i.e. the fittest trait in the absence of DIPs. I think this part could benefit from the addition of a plot of $\bar{x}_v$ in fig 7, as that is the quantity being referenced, but never shown.

8. Even though the phenotype is quite abstract, the authors highlight a strong theoretical conclusion in lines 395-402: I think this should be re-proposed in the discussion, better indicating what this might mean experimentally. This would strengthen the conclusions and impact of the paper.

9. In the discussion, there is space to refer to the fact that these results could be studied analytically. This is particularly possible given the choice of quadratic-in-x fitness functions and the conclusion that changes in μ (getting smaller and smaller) do not seem to impact the evolutionary outcome (fig 5F). Thus an asymptotic study, typical of these models, could be conducted (or has this already been conducted by Alfaro and co-workers? If so, appropriate reference and merit should be given). Additional citations would also improve the mathematical impact of the paper.

10. Lines 444-448: what are these orders of magnitude? A more precise comment of the scales of dynamics would be useful. Wouldn't changing the order of magnitude of μ suffice to capture

this?

Minor points

1. In the abstract it is far from clear what the trait represents. This is touched upon in the author summary, but I would recommend including it also in the abstract for clarity of context.

2. The literary context is really well-written and clear, but i would recommend adding a paragraph on the mathematical context of phenotype-structured PDE models, which is very vast.

3. I would recommend writing $I_v[d](x,t)$ and $I_d[v](x,t)$ since these functions are defined as operators on d and v: this would be more precise mathematically and better highlight the nonlinear coupling of the equations.

4. I find the content of lines 119-112 unnecessary, it suffices to say that identity (4) si a direct consequence of the definitions (1) and (2). Moreover, I believe the content of lines 122-128 would be better conveyed by mentioning the use of different κ in (1) and (2). I would introduce κ_v and κ_d in (1) and (2), later saying that for [the reasons given] for now we choose $\kappa_v = \kappa_d \equiv \kappa$ – but this is just a suggestion based on personal preference, the authors should feel no obligation to implement this is it does not suit their style.

5. Typo in (6), should be L(t).

6. I don't get the point of the numbered scenarios in figure 1 right panel: are they relevant to the setup or conclusions?

7. The authors consider two intial setups for the simulations, either with DIPs starting with the same mean trait as the virus or far from it: this should be better motivated since the authors state in the model that DIPs can emerge but with the same trait as the virus.

8. I am not sure of the relevance of fig 4A and 4B: this seems to repeat information we already saw in previous figures.

9. For the global sensitivity analysis, the distribution of the parameters is missing (I assume truncated uniform, but this should be specified as it may impact the outcomes).

10. Lines 303-304: when stating the "likelihood of each regime" it would be helpful to clarify that this is intended as a proportion of scenarios (this comes out only when using %).

11. Line 388-389: Rast et al. which year? Ref missing.

12. In the discussion, the authors propose relevant model modifications for increased biological relevance. I think an important extension to discuss here is the use of a nonlocal mutation kernel in place of a diffusion operator. In fact, the authors talk about mutations, giving examples of phenotypic changes which could result in jumps in phenotype space, rather than small variations. This would also be consistent with the initial condition considering the mean DIP trait to be far from the mean virus trait. This could generalise the model and include further levels of biological complexity. Many structured PDE models consider nonlocal changes, it should not be hard to find an appropriate reference.

13. Finally, some figures (2, 3 and maybe also 4) could benefit from titles to plots to distinguish between top and bottom rows. The surfplots of figure 4C are a bit faded, a sharper color would help.
