# Supplementary material for: Coevolutionary dynamics of viruses and their defective interfering particles
Source: PLoS Comput Biol. 2026 May 20;22(5):e1014300. doi: 10.1371/journal.pcbi.1014300 (PMC13232958; doi:10.1371/journal.pcbi.1014300)
Supplement: Attachment — Submitted filename: Muthupandiyan Responses to Reviewers April2026.pdf [file pcbi.1014300.s010.pdf]

Response to Reviewers
PCOMPBIOL-D-25-02183
Title: Coevolutionary dynamics of viruses and their defective interfering particles
Authors: Muthupandiyan and Yin

Reviewer 1

Major points

1. I found the model description tricky to follow since the authors introduce individual terms in the PDEs before introducing the PDEs. This was only made easier by the fact that I am very familiar with phenotype-structured PDE models, and I think a change in presentation may better guide readers interested in the application who may not be too familiar with this type of models.

As the reviewer suggested, we restructured the Methods section to present the governing PDE system (Eqs 1–2) at the outset, followed by a term-by-term explanation of each component. This gives readers an immediate overview of the full model before the detailed derivations.

2. In figure 1 the authors show an experimental setup: this is interesting and I think it should be better introduced in the manuscript, together with appropriate references. For instance I am not sure whether it is an illustration of the abstraction level of the model, or an actual setup used in wet lab experiments. If the latter is the case, than this is a point in favour for the paper which would better tie together the biological conclusions. In fact, I think the use of titled paragraphs in the discussion could be useful, with one dedicated to the interdisciplinary conclusions and perspectives.

We substantially expanded the connection between the model and experimental systems. The Methods now include a new paragraph explicitly identifying the continuous-culture bioreactor as the experimental system most directly realized by our assumptions, citing Frensing et al. (2013) for continuous influenza propagation and explaining how serial passaging (von Magnus, 1951; DePolo et al., 1987) represents a discrete analog. We also revised the Fig 1 caption to better describe the connection between the experimental setup and the abstract phenotype space.

The Discussion now uses titled paragraphs throughout: “Parameter Validation,” “Model Limitations,” “Analytical Tractability,” “Experimental Predictions,” and “Therapeutic Implications.” The Experimental Predictions subsection draws together the interdisciplinary conclusions.

3. A critical point is that the authors in the results section often discuss the numerical outcomes talking about “stable” dynamics reached by the system, e.g. line 261 “a stabe, cyclical pursuit”. How is stability determined numerically? For instance while in fig 3E it looks like a steady dynamics has been reached, the associated population dynamics in fig 3D do not appear to have stabilised, suggesting this might be a transient behaviour. The authors should better indicate how certain conclusions are quantitatively determined.

We agree that our use of “stable” was imprecise. We added an explicit statement in the Numerical Approach section clarifying that references to “stable” or “sustained” dynamics describe behavior observed over the second half of the simulation window, and that we do not claim formal mathematical stability. Regarding Fig 3D specifically, we added a note to the caption acknowledging that the

population dynamics may not have fully equilibrated; the cyclical pursuit visible in Fig 3E is what we characterize as sustained, not the transient population oscillations. We also added a convergence analysis (Table S3) comparing classifications at T = 200 and T = 400, showing 93% concordance.

4. On a similar note, the authors should better state how an "outcome" is determined. For instance in the sensitivity analysis, are they plotting the observed dynamics at a certain fixed time? If so, how is this time chosen? Indeed the convergence speed to a steady outcome may change as parameter values change. How is convergence to an outcome determined numerically? The same question holds for the results of fig 7: what is meant by "final" virus population? This is even more vague if the system stabilises to a cyclic solution of coevolutionary chase, in which case population numbers will fluctuate in time. What summary statistic is being plot exactly? What were the initial conditions for these simulations? This improved precision is required throughout the results section.

To respond to the reviewer's questions, we substantially expanded the description of our classification methodology in the Sensitivity Analysis subsection. We now explicitly state: (1) all simulations ran to a fixed end time of T = 200; (2) outcomes were assessed using only the second half of each simulation to reduce sensitivity to transient dynamics; (3) Coextinction was defined as the virus population falling below 10^{-6}; (4) DIP Extinction was defined as the DIP population falling below 10^{-6} while the virus persisted; (5) Chase was classified when the standard deviation of the phenotypic distance $\Delta(t)$ exceeded 0.1 over the assessment window; and (6) Coexistence otherwise.

For Fig 7 (new Figure 6), we now specify that the "final virus population" is the time-averaged total virus population over the final 20% of each simulation, normalized by carrying capacity and plotted on a logarithmic scale. The "escape extent" is the time-averaged Euclidean distance between the virus centroid and the fitness optimum. Initial conditions are stated in the figure caption: virus and DIP populations of 10^6 each, centered at (1, 1) and (1.4, 0), respectively.

5. The (mathematical) definition of "phenotypic distance" should be explicitly stated, indicating what summary statistic is being plot in fig 4b. Also, here the authors use the notation ¯xD but never introduced it before – this could be done in (9).

We added an explicit definition of xD alongside xV (new Eqs 10, 11). We also added a new "Summary Statistics" passage formally defining phenotypic distance as the Euclidean distance between virus and DIP centroids (new Eq 12).

6. In fig 6 and Cyclical escape: it is not clear how "resistance" is defined and computed. A (mathematical) definition of the quantity plot is necessary. I find the visualisation in this figure very confusing, especially since we are looking at a function of time and time.... If I have inferred correctly, this is again the distance between ¯xv(t) and ¯xd(t), seen as a function of x̄v(t) and ¯xd(t) which vary on the x- and y-axis respectively. I understand that the authors are choosing to compare "resistance" (i.e. phenotypic distance?) of the virus to DIPs at the same time or in the past or future to reproduce this concept of transient resistance found in the experimental paper cited. What is not clear to me is why this is relevant: since the dynamics of virus and DIPs are coupled, we will never actually have to deal with the current phenotype of the virus interacting with the past or future phenotype of DIPs. I am certain DePolo et al. gave a relevant motivation for this, which should be indicated, along with how the quantities plot in fig 6C were defined, obtained and measured experimentally.

We added a formal definition of fold resistance R in the Methods (new Eq 14). Normalized interference measures the per-capita interference a virus snapshot would experience from a given DIP snapshot, computed from population-normalized densities to remove dependence on population size. Fold resistance is the ratio of the maximum interference any virus snapshot experiences from a given DIP to the interference experienced by the focal virus, analogous to fold-resistance assays used experimentally. For clarity, we also unified the terminology throughout the manuscript under "fold resistance," eliminating the previous inconsistent use of "fold reduction."

Regarding relevance: we added a clarifying passage noting that although virus and DIP populations only ever interact contemporaneously during infection, time-shift experiments (in which historical isolates are crossed against a fixed DI particle stock) are a standard diagnostic tool for distinguishing modes of coevolution (Gaba & Ebert, 2009). We also expanded the Fig 6 (new Fig 5) caption to describe the original experimental protocol of DePolo et al.

7. The results of simulations plot in fig 7 are often referring to how close the trait of the virus is to the origin, i.e. the fittest trait in the absence of DIPs. I think this part could benefit from the addition of a plot of $\bar{x}v$ in fig 7, as that is the quantity being referenced, but never shown.

We added a new panel (old Fig 7C, new Fig 6C) showing time series of the virus centroid distance from the fitness optimum ($\|\bar{x}_V\|$) and the distance between virus and DIP centroids ($\|\bar{x}_V - \bar{x}_D\|$) for three representative values of α at fixed κ. This directly visualizes the trapped, intermediate, and escape regimes referenced in the text.

8. Even though the phenotype is quite abstract, the authors highlight a strong theoretical conclusion in lines 395-402: I think this should be re-proposed in the discussion, better indicating what this might mean experimentally. This would strengthen the conclusions and impact of the Paper.

We added a new “Experimental Predictions” subsection to the Discussion that reproposed this finding and connected it to specific experimental tests. We now state that competition assays quantifying the fitness cost of known resistance mutations, combined with deep sequencing, could place a specific virus–DIP pair on the α–κ landscape of Fig 7 (new Fig 6) and inform whether a TIP strategy requires additional design constraints to prevent evolutionary escape.

9. In the discussion, there is space to refer to the fact that these results could be studied analytically. This is particularly possible given the choice of quadratic-in-x fitness functions and the conclusion that changes in μ (getting smaller and smaller) do not seem to impact the evolutionary outcome (fig 5F). Thus an asymptotic study, typical of these models, could be conducted (or has this already been conducted by Alfaro and co-workers? If so, appropriate reference and merit should be given). Additional citations would also improve the mathematical impact of the paper.

We added a new “Analytical Tractability” subsection to the Discussion. We note that the model’s structural choices are shared with systems that have been studied analytically, including the replicator-mutator equation where Gaussian distributions are preserved exactly (Chisholm et al. 2016; Alfaro & Carles 2014, 2017) and the host-pathogen system of Alfaro et al. (2025), where traveling-pulse solutions were proven to exist. We discuss why direct application to our coupled system is not straightforward (the interference convolution introduces non-quadratic effective fitness terms, and de novo generation

can produce bimodal DIP distributions) and identify directions for future analytical work including moment closure and Hamilton-Jacobi asymptotics.

We also expanded the Introduction with a new paragraph providing mathematical context for phenotype-structured PDE models, citing Dieckmann & Law (1996), , Perthame & Barles (2008), Chisholm et al. (2016) , and others.

10. Lines 444-448: what are these orders of magnitude? A more precise comment of the scales of dynamics would be useful. Wouldn't changing the order of magnitude of μ suffice to capture this?

As suggested by the reviewer, we now quantify the discrepancy explicitly (see revision lines 586-599): the modeled resistance changes span roughly 10–50-fold, whereas the experimental data of DePolo et al. (1987) show resistance peaks of 10^5-fold. We attribute this primarily to the two-dimensional phenotype space, which limits the maximum phenotypic separation the virus can sustain against the centering penalty (α). Fold resistance is determined by the overlap between virus and DIP distributions through the convolution integrals, and this overlap decays as a Gaussian with separation. Increasing the accessible dimensionality would substantially amplify fold-resistance values by allowing greater separation, consistent with the model's extension to three dimensions (Fig S4).

Regarding whether changing μ could recover the experimental magnitudes: the sensitivity analysis (new Fig 4F) shows that μ has negligible influence on outcomes across the examined range, indicating that the binding constraint is the dimensionality of the phenotype space rather than the diffusion rate. The bounding of the centering penalty (α) prevents the virus from travelling sufficiently far from the DIP to recreate the resistance peaks observed in DePolo.

Minor points
1. In the abstract it is far from clear what the trait represents. This is touched upon in the author summary, but I would recommend including it also in the abstract for clarity of context.

The abstract now includes the phrase "across continuous traits (e.g. replicase binding affinity or packaging signal strength)" to clarify the nature of the phenotype space.

2. The literary context is really well-written and clear, but i would recommend adding a paragraph on the mathematical context of phenotype-structured PDE models, which is very vast.

As noted in point (9) above, we added a paragraph to the Introduction providing more mathematical context for phenotype-structured PDE models.

3. I would recommend writing Iv[d](x, t) and Id[v](x, t) since these functions are defined as operators on d and v: this would be more precise mathematically and better highlight the nonlinear coupling of the equations.

As suggested, the interference terms are now written as IV[d](x, t) and ID[v](x, t) throughout the manuscript and in the parameter table

4. I find the content of lines 119-112 unnecessary, it suffices to say that identity (4) si a direct

consequence of the definitions (1) and (2). Moreover, I believe the content of lines 122-128 would be better conveyed by mentioning the use of different κ in (1) and (2). I would introduce κv and κd in (1) and (2), later saying that for [the reasons given] for now we choose κv = κd ≡ κ – but this is just a suggestion based on personal preference, the authors should feel no obligation to implement this is it does not suit their style.

As the reviewer suggested, we now introduce κV and κD separately in the interference definitions (new Eqs 3, 4) and then state that we set them equal to represent a zero-sum resource transfer. We also shortened the conservation identity passage (new Eq 6) to note that it follows directly from the symmetry of the convolution integrals, removing the explicit derivation.

5. Typo in (6), should be L(t).

Corrected (new Eq 7).

6. I don't get the point of the numbered scenarios in figure 1 right panel: are they relevant to the setup or conclusions?

We revised the Fig 1 caption to remove the numbered scenarios and replace them with a clearer description focused on the central tradeoff the model explores: whether the virus remains near the fitness optimum despite strong interference from co-localized DIPs, or displaces from it to reduce interference at the cost of lower intrinsic fitness.

7. The authors consider two intial setups for the simulations, either with DIPs starting with the same mean trait as the virus or far from it: this should be better motivated since the authors state in the model that DIPs can emerge but with the same trait as the virus.

We added a passage (revision lines 360-366) explaining that the separated initialization models the practically relevant scenario of introducing a DIP or therapeutic interfering particle into a culture with an established virus population.

8. I am not sure of the relevance of fig 4A and 4B: this seems to repeat information we already saw in previous figures.

We removed the redundant panels and incorporated Fig 4 (new Fig 3), now consisting solely of the heatmap snapshots showing population distributions in phenotype space at sequential time points, with a revised caption.

9. For the global sensitivity analysis, the distribution of the parameters is missing (I assume truncated uniform, but this should be specified as it may impact the outcomes).

We now explicitly state the sampling distributions used in the LHS analysis: log-uniform for all log-scaled parameters and uniform for γ. Details are in the clean revision (lines 394-402).

10. Lines 303-304: when stating the "likelihood of each regime" it would be helpful to clarify that this is intended as a proportion of scenarios (this comes out only when using %).

Changed to "proportion of simulations in each regime."

11. Line 388-389: Rast et al. which year? Ref missing.

Added the year and reference: Rast et al. (2016).

12. In the discussion, the authors propose relevant model modifications for increased biological relevance. I think an important extension to discuss here is the use of a nonlocal mutation kernel in place of a diffusion operator. In fact, the authors talk about mutations, giving examples of phenotypic changes which could result in jumps in phenotype space, rather than small variations. This would also be consistent with the initial condition considering the mean DIP trait to be far from the mean virus trait. This could generalise the model and include further levels of biological complexity. Many structured PDE models consider nonlocal changes, it should not be hard to find an appropriate reference.

We added a new paragraph to the Discussion proposing the replacement of the diffusion operator with a nonlocal mutation kernel as a natural generalization. We cite Alfaro (2022) for confining nonlocal kernels and Champagnat et al. (2006) for the derivation of the Laplacian as a specific scaling limit of integral operators.

13. Finally, some figures (2, 3 and maybe also 4) could benefit from titles to plots to distinguish between top and bottom rows. The surfplots of figure 4C are a bit faded, a sharper color would help.

We added row labels (“De Novo Emergence” and “Initial DIP Population”) to figures 2 and 3. We increased the color saturation in the Fig 4 (new Fig 3) heatmaps.

Reviewer 2

1) Equations (12,13) are almost identical to equations (10,11). The only difference is that the parameter rV is replaced by 1, a scaling that could easily have been done at the start by suitably defining the time units. Thus it seems that there is some unnecessary work towards the end of the Methodology section, which hopefully the authors can streamline.

Following the suggestion by Reviewer 1, we now present the full dimensional model in Equations 1 and 2. The non-dimensionalized form of the model is now presented in the Supplemental Information (S7)

2) At the bottom of page 7 some specific numerical values are selected. Why these numbers in particular/ how robust are results if these are changed? We see later in the paper what happens when single parameter values are varied in turn, but what if a completely new set of parameters was used?

In response to Reviewer 2, we have now added a "Parameter selection and biological grounding" subsection to the Results that provides explicit justifications for each baseline parameter, grounded in experimental measurements where available. We also clarified that the Latin Hypercube Sampling sensitivity analysis draws each of the 10,000 simulations from a unique combination of all six parameters simultaneously (not one at a time), effectively testing 10,000 distinct parameter sets. The stacked proportion plots show marginal trends that are robust to simultaneous variation in all other parameters across their full ranges. This directly addresses the concern about robustness to entirely new parameter sets. In the Discussion, the new “Parameter Validation” subsection presents macroscopic validation through correspondence with experimentally observed phenomena.

3) In Figure 6 and its associated caption, the terms "fold reduction" and "fold resistance" are used. These are mentioned nowhere else, however, and not explained. What do they mean and are these different things? What is their relevance?

As addressed above (Reviewer 1, Major Point 6), we added explicit mathematical definitions of this quantity in the Methods (new Eq 14) and unified the terminology under "fold resistance" throughout the manuscript and figure captions. Fold resistance of 1 indicates maximal susceptibility, while higher values indicate greater resistance. This measure is analogous to fold-resistance assays used in experimental time-shift studies.

4)In Figure 7 A, can the authors explain the meaning of the line crossings, and why they occur?

The minor crossings (new Fig 6A) between adjacent α curves (e.g., α = 1.0 vs. 0.5) are not robust to changes in the simulation time horizon or averaging window, and reflect sensitivity to transient dynamics in specific simulations rather than distinct mechanistic regimes. They arise because the time-averaged population in the assessment window can be influenced by the phase of ongoing oscillations at the time of measurement.

Editor

In particular, it is not clear how the model parameters were chosen, nor how these could be inferred from the experimental system described in Figure 1. As many of the results in this manuscript are obtained via model simulation, it is extremely important to justify where these parameters are coming from, and why they are appropriate, though I appreciate that the authors have performed a 1 by 1 sensitivity analysis. This is particularly relevant to ensure that the conclusions drawn in this manuscript drive new biological understanding, rather than mathematical artifacts of the model framework chosen. Consequently, it will be important to fully address Reviewer 2's 2nd point.

We appreciate the Editor's emphasis on establishing that our model-derived conclusions reflect biological reality. We have addressed this through three complementary revisions.
First, we added a "Parameter selection and biological grounding" subsection to the Results, providing explicit justifications for each baseline parameter value. Where direct experimental measurements exist, we cite them and show consistency with our chosen values. Where direct grounding is not possible, we provide qualitative arguments for the chosen order of magnitude and demonstrate via the sensitivity analysis that the model's qualitative conclusions are robust to variation in these parameters.
Second, we reorganized the Discussion to include a dedicated "Parameter Validation" subsection that presents two forms of macroscopic validation: (a) the model's emergent dynamics reproduce qualitative phenomena observed in experimental systems), and (b) the global sensitivity analysis demonstrates that the qualitative regimes persist across simultaneous variation of all parameters over orders of magnitude, confirming they are not artifacts of a single parameter set.
Third, we added an “Experimental Predictions” subsection to the Discussion proposing specific experiments that would enable direct calibration of the model’s abstract parameters, including competition assays for fitness costs (α), controlled co-infection experiments at varying multiplicities for interference strength (κ), and bioreactor experiments with defined dilution rates for comparative tests of the model’s predictions about washout effects (γ).

Similarly, it is important to further justify the modelling decisions made when developing the phenotypically structured model. While the current modelling choices may allow for an analytical investigation of the model (as reviewer 1 notes), as the model is not validated against experimental data, it is increasingly important to either strongly justify these modelling choices or show that the biological conclusions drawn from this model are robust to alternative model formulations.

We have strengthened the justification of our core modeling choices in multiple ways. The quadratic fitness landscape is now justified as the leading-order Taylor approximation of any smooth fitness surface near an isolated optimum, making it the generic local approximation independent of specific biological mechanism. The robustness of our biological conclusions to alternative model formulations was already partially demonstrated in the supplementary material (alternative interference kernels in Fig S5, extension to three trait dimensions in Fig S4), and we now discuss these more prominently in a new “Parameter Validation” subsection. We also added discussion of nonlocal mutation kernels as a natural generalization.

Finally, I note PLOS Computational Biology's policy on the use of artificial intelligence (https://journals.plos.org/plosone/s/ethical-publishing-practice#loc-artificial-intelligence-tools-and-technologies) : Contributions by artificial intelligence (AI) tools and technologies to a study or to an article’s contents must be clearly reported in a dedicated section of the Methods, or in the Acknowledgements section for article types lacking a Methods section. This section should include the name(s) of any tools used, a description of how the authors used the tool(s) and evaluated the validity of the tool’s outputs, and a clear statement of which aspects of the study, article contents, data, or supporting files were affected/generated by AI tool usage

We have added a section to the Methods section describing the use of LLMs for debugging code and initial visualizations.
